# *ESR*α Promoter Methylation May Modify the Association Between Lipid Metabolism and Type 2 Diabetes in Chinese Farmers

**DOI:** 10.3389/fpubh.2021.578134

**Published:** 2021-03-04

**Authors:** Guoyu Zhou, Lihua Liu, Xing Li, Xiangbo Hou, Ling Wang, Renjie Sun, Hui Huang, Zhiyuan Li, Wenjie Li, Chongjian Wang, Yue Ba

**Affiliations:** ^1^Department of Environment Health & Environment and Health Innovation Team, School of Public Health, Zhengzhou University, Zhengzhou, China; ^2^Yellow River Institute for Ecological Protection & Regional Coordinated Development, Zhengzhou University, Zhengzhou, China; ^3^Department of Nutrition and Food Health, School of Public Health, Zhengzhou University, Zhengzhou, China; ^4^Department of Epidemiology and Biostatistics, School of Public Health, Zhengzhou University, Zhengzhou, China

**Keywords:** estrogen receptor alpha, DNA methylation, lipid metabolism, diabetes mellitus, farmers

## Abstract

**Objective:** This study is aimed to explore the potential association among the estrogen receptor alpha (*ESR*α) promoter methylation, lipid metabolism and the risk of type 2 diabetes mellitus (T2DM).

**Methods:** A total of 1143 rural residents were recruited randomly from Henan Province, China. The circulating methylation levels in *ESR*α promoter region were determined by quantitative methylation-specific polymerase chain reaction. Serum high density lipoprotein cholesterol (HDL-C), low density lipoprotein cholesterol (LDL-C), triglyceride (TG), total cholesterol (TC) and fasting plasma-glucose (FPG) were measured.

**Results:** The *ESR*α promoter methylation levels were negatively associated with HDL-C levels whether gender stratification was performed (*P* < 0.05) and positively correlated with LDL-C in men (*P* < 0.05). Each unit standard deviation (SD) increment in TG was associated with a 43% increase (95% CI: 1.25, 1.64) in the risks of T2DM in all participants, a 36% increase (95% *CI*: 1.13, 1.64) in the risks of T2DM in men and a 49% increase (95% CI: 1.21, 1.83) in the risks of T2DM in women. Furthermore, each SD increment in HDL-C was associated with a reduction of 25% (OR = 0.75, 95% CI: 0.58, 0.97) in the risks of T2DM in men, and the risk of T2DM in men may be more susceptible to HDL-C than that in women (*P* for interaction < 0.05). Additionally, we found that the risk of T2DM in participants with lower methylation levels (≤4.07%) were more susceptible to HDL-C (*P* for interaction < 0.05).

**Conclusions:** These findings suggested that lipid metabolism was associated with *ESR*α promoter methylation levels and the risk of T2DM. Besides, the levels of *ESR*α promoter methylation and gender can modify the association of HDL-C and T2DM.

## Background

Type 2 diabetes mellitus (T2DM) poses a worldwide public health problem with a continuously increasing prevalence in both developing and developed countries (1–3). More than half of patients with T2DM suffer from dyslipidemia (4). Diabetic dyslipidemia is mainly a mixed dyslipidemia with higher triglycerides (TG) and low density lipoprotein cholesterol (LDL-C), and lower of high density lipoprotein cholesterol (HDL-C) which can be observed before the onset of diabetes (5–7). The use of lipid-lowing therapy can improve lipid metabolism and prevent T2DM (8). Consequently, exploring the molecular mechanisms underlying abnormal lipid metabolism and T2DM pathogenesis is critical to develop therapeutic strategies for T2DM.

Several evidences indicate that estrogens can regulate lipid metabolism and protect mouse from β-cell apoptosis (9, 10). Estrogens deficiency can contribute to metabolic dysfunction, and then cause obesity and insulin resistance (11, 12). Additionally, the estrogens therapy has been revealed to have various beneficial effects by decreasing fasting glucose, increasing insulin sensitivity and secretion and reduce T2DM incidence in postmenopausal women (13, 14). Notably, the metabolic effects of estrogens are mediated by estrogen receptor alpha (ESRα) (15). Animal studies revealed that mice were more resistant to insulin after *ESR*α knockout (11). Furthermore, Ribas et al. (16) found that ESRα deficiency can increase fasting insulin levels, impairs glucose tolerance and results in skeletal muscle insulin resistance. As an important epigenetic modification, DNA methylation is a key regulator of gene expression. Additionally, the methylation of *ESR*α promoter is reported to reduce the expression of ESRα (17). Furthermore, abnormal DNA methylation were found to be associated with lipid metabolism disorders (17, 18). However, whether the alteration of *ESR*α promoter methylation affects human lipid metabolism and the risk of T2DM has not been explored.

Given these, in this study, we conducted a cross-sectional study in rural areas of Henan Province, and recruited 1,143 Chinese farmers to identify the association of *ESR*α promoter methylation, lipid metabolism and T2DM. With the development of targeted interventions for DNA methylation (19), this study can provide a theoretical basis for the screening of diabetes-susceptible populations and future precision therapy.

## Methods

### Study Participants

A cross-sectional study was conducted in Wuzhi County of Henan Province in China by random sampling in 2013. Participants were excluded as the following: (1) people with type 1 diabetes; (2) secondary diabetes (drug-induced, chemical-induced, exocrine pancreatic insufficiency, and genetic defects); (3) gestational diabetes and rare forms of diabetes. Finally, a total of 1,143 local permanent residents were recruited in this study. The project was approved by the Institutional Review Board at Zhengzhou University. All participants were informed of the purpose of the study and provided written informed consent.

### Outcome Variable

The diagnostic testing for T2DM was performed according to the criteria of World Health Organization (1999) (20) and the guidelines of American Diabetes Association (2002). All the nondiabetic people had normal glucose tolerance after an oral glucose tolerance test (OGTT).

### Sampling and Data Collection

Trained investigators conducted a face-to-face interview with each participant using a standard questionnaire for obtaining information of demographic characteristics including age, gender, economic status, educational level, dietary habits and lifestyle (smoking and drinking, salt intake, physical activity, et, al). Among participants, those who had smoked at least 100 cigarettes in their lifetime were defined as “smoking”; those consuming any drink containing alcohol more than 12 times during the past 12 months were defined as “drinking.” A total of 10 mL fasting blood samples (5 ml of anticoagulative and 5 ml of non-anticoagulative) were collected from each participant. Serum samples were isolated from non-anticoagulative blood after centrifugation (3,000 rpm for 15 min) at 4°C and frozen at −80°C for subsequent analyses.

### Measurement of Biochemical Parameters

The concentration (mmol/L) of HDL-C, LDL-C, TG, total cholesterol (TC) and FPG in serum samples were measured with direct method of catalase clearance, direct method of surfactant removal, glycerol phosphate oxidase-peroxidase (GPO-PAP), cholesterol oxidase-peroxidase (GHOD-PAP) and glucose oxidative method, respectively (21). Finally, 120 serum samples were randomly selected for repeated measurements. All analyses were run on an automatic biochemical analyzer (Kehua Bio-engineering Co., Ltd, Shanghai, China).

### Measurement of *ESRα* Promoter Methylation

The genomic DNA was extracted from whole blood samples using a BioTeke Magnetic beads kit (Bioteke Crporation, Beijing, China). The concentration of DNA samples was measured using a Nanodrop ND-2000 spectrophotometer (Thermo, MA, USA). Subsequently, the genomic DNA were treated with sodium bisulfite using an EZ DNA Methylation-Gold kit (Zymo Research, CA, USA). The putative promoter sequences of *ESR*α the sequence of the gene promoter region assumed to be 2,000 bp upstream from the *ESR*α start codon (22)] were obtained from UCSC/Ensembl, and then primer sequences (methylated specific primers: L, 5′-CGT AGG TTT ACG GTT AGA TCGG-3′; R, 5′-ATA CAA TAA CAT CAA CGA ACT CGAA-3′; unmethylated specific primers: L, 5′-ATG GTT AGA TTG GTT TTT TTT TAGG-3′; R, 5′-ACA TCA ACA AAC TCA AAA ACA CACT-3′) were designed using the methylation primer design software (Methyl Primer Express v1.0). The *ESR*α methylation level was analyzed using quantitative methylation-specific PCR on a MX3000P real-time PCR system (Aglient, Santa Clara, CA, USA). PCR amplification was performed in a 15 μl reaction mixture contained 5.5 μl of diluted DNA template (100 ng), 7.5 μl of 2 × Power SYBR Green PCR Master Mixture (CWBIO, Beijing, China), 2 μl of primer with a concentration of 1.25 μmol/L each. The PCR cycling parameters were as follows: 95°C for 10 min; 40 cycles for degeneration at 94°C for 15 s, annealing at 54°C for 30 s, and extension at 72°C for 30 s. Two negative controls (replace the DNA template with ddH_2_O) were set for each plate. The level of DNA methylation was calculated according to the formula: [1/(1+2^−ΔCt^)]×100%, where Δ*Ct*=Ct(unmethylated)–Ct(methylated) (23). Ct is the threshold of PCR cycle number at which the increase in fluorescent signal reaches a critical point. Each sample was analyzed in duplicate.

### Statistical Analysis

The Student's *t*-test, Wilcoxon test and Chi-square test were used to analyze the differences in normal/near-normal characteristics, other continuous variables and categorical variables between participants in T2DM group and non-diabetic group. We then utilized linear regression model to exam the association between *ESR*α methylation level and lipid metabolism. Besides, the association between T2DM and lipid metabolism (as well as *ESR*α methylation level) were estimated using binary logistic regression model. The linear trends across increasing quartiles of *ESR*α methylation level and lipid metabolism were estimated by treating the median of each quartile as a continuous variable. The quartiles of *ESR*α methylation and lipid metabolism are shown in Supplementary Table 1. After stratifying the participants according to the median of *ESR*α promoter methylation level, we analyzed the interactive effect of *ESR*α methylation and lipid metabolism on the risk of T2DM by adding an interaction term “*ESR*α methylation ^*^ lipid metabolism” to the logistic regression model. In addition, we performed other interactive analyses by adding an interaction term (“*ESR*α methylation^*^gender” or “lipid metabolism^*^gender”) to each models. According to the characteristics of the participants and the previous reports, we adjusted a variety of potential confounding variables (including age, gender, BMI, educational level, smoking, drinking and household income) in this study.

All statistical analyses were performed by SPSS 22.0 (IBM Corp, Armonk, NY, USA). The *P*-values < 0.05 were considered statistically significant.

## Results

### Distribution of Variables in Different Groups

The characteristics of the 1,143 residents are summarized in Table 1. As compared to the non-diabetic group, the T2DM group have a lower proportion of men/women, drinking and vegetables intake (≥500 g/day), and a higher proportion of illiteracy and family diabetes history (*P* < 0.05). Besides, the average age, BMI, TG and TC in T2DM group were higher than those in non-diabetic group (*P* < 0.05). The distributions of household incomes, *ESR*α promoter methylation, HDL-C and LDL-C are comparable between the two groups.

**Table 1:** Characteristics of participants in T2DM group and Nondiabetic group^a^.

**Characteristics**	**T2DM group**	**Nondiabetic** **group**	***t*/*χ^2^*/*Z***	***P***
	**(*n* = 237)**	**(*n* = 906)**		
Age (years)	57.58 ± 8.96	54.50 ±10.08	4.588	<0.001
Gender			6.195	0.013
Men	103 (43.5)	476 (52.5)		
Women	134 (56.5)	430 (47.5)		
BMI (kg/m^2^)	26.35 ± 3.70	25.62 ± 3.58	2.785	0.005
Education level			10.951	0.012
Illiteracy	51 (21.6)	121 (13.4)		
Primary school	65 (27.5)	246 (27.2)		
Junior high school	93 (39.4)	406 (44.9)		
High school and above	27 (11.4)	131 (14.5)		
Smoking			1.875	0.171
Yes	75 (31.6)	330 (36.4)		
No	162 (68.4)	575 (63.6)		
Drinking			6.040	0.014
Yes	34 (14.3)	195 (21.5)		
No	203 (85.7)	711 (78.5)		
Vegetables intake (g/day)			8.728	0.003
≥500	56 (23.7)	306 (33.8)		
<500	180 (76.3)	600 (66.2)		
Household income (RMB/year)			0.826	0.662
<6,000	182 (76.8)	670 (74.0)		
6,000–12,000	43 (18.1)	182 (20.0)		
>12,000	12 (5.1)	54 (6.0)		
Family diabetes history		5.869	0.015
Yes	52 (22.2)	140 (15.6)		
No	182 (77.8)	760 (84.4)		
*ESRα* methylation (%)	4.58 ± 2.22	4.53 ± 2.21	0.338	0.735
FPG (mmol/L)	8.65 (6.68, 10.99)	4.81(4.33, 5.34)	20.08	<0.001
TG (mmol/L)	2.28 ± 1.67	1.72 ± 1.29	4.746	<0.001
TC (mmol/L)	4.77 ± 1.05	4.58 ± 0.97	2.558	0.011
HDL-C (mmol/L)	1.22 ± 0.31	1.24 ± 0.30	0.653	0.514
LDL-C (mmol/L)	2.59 ± 0.83	2.59 ± 0.75	0.099	0.922

*RMB, China Yuan*.

a *Data are expressed as the mean ± SD or Median (P25, P75) for continuous variables and n (%) for categorical variables*.

After stratifying the participants according to the tertiles of age, we found that found that the levels of *ESR*α methylation, FPG and LDL-C in the elderly were elevated (all *P* < 0.05). There is no statistically significant difference in the levels of TG, TC, HDL-C among different groups (Supplementary Table 2).

### Association Between *ESRα* Methylation Level and Lipid Metabolism

As shown in Table 2, the HDL-C level showed a downward trend with the increase of the *ESR*α promoter methylation level regardless of gender stratification (*P* for trend < 0.05). Besides, we found that in the continuous analysis, the *ESR*α promoter methylation level was negatively associated with HDL-C levels whether gender stratification was performed (all *P* < 0.05). For each unit standard deviation (SD) increment in *ESR*α promoter methylation, the level of HDL-C decreased by 0.03 mmol/L in all participants, 0.04 mmol/L in men and 0.03 mmol/L in women. Besides, we observed an increase of 0.08 mmol/L of LDL-C in men with each unit SD increment in *ESR*α promoter methylation level (*P*=0.037).

**Table 2:** Association between *ESR*α methylation and lipid metabolism.

***ESRα* methylation** **(%)**	**All**^****^a^****^		**Men**^****^b^****^		**Women**^****^b^****^		***P* for interaction^**^c^**^**
	**β (95% CI)**	***P***	**β (95% CI)**	***P***	**β (95% CI)**	***P***	
**TG (mmol/L)**							
Quartile 1	Reference		Reference		Reference		
Quartile 2	−0.07(−0.31, 0.17)	0.580	−0.25(−0.63, 0.12)	0.185	0.17(−0.12, 0.46)	0.257	
Quartile 3	0.27(0.02, 0.51)	0.037	0.01(−0.37, 0.35)	0.974	0.48(0.15, 0.81)	0.005	
Quartile 4	−0.04(−0.26, 0.17)	0.007	−0.20(−0.55, 0.15)	0.262	0.06(−0.19, 0.31)	0.642	
Trend test		0.907		0.511		0.759	
Increase per SD	−0.03(−0.12, 0.05)	0.461	−0.07(−0.21, 0.06)	0.297	−0.02(−0.13,0.08)	0.659	0.258
**TC (mmol/L)**							
Quartile 1	Reference		Reference		Reference		
Quartile 2	−0.09(−0.27, 0.08)	0.297	−0.13(−0.38, 0.13)	0.321	0.01(−0.22, 0.25)	0.906	
Quartile 3	0.08(−0.10, 0.27)	0.379	0.02(−0.25, 0.28)	0.913	0.16(−0.09, 0.41)	0.220	
Quartile 4	−0.07(−0.24, 0.10)	0.425	−0.06(−0.33, 0.21)	0.651	−0.10(−0.32, 0.12)	0.381	
Trend test		0.781		0.952		0.411	
Increase per SD	−0.01(−0.07, 0.06)	0.838	0.01(−0.09, 0.10)	0.851	−0.04(−0.12, 0.04)	0.340	0.951
**HDL-C (mmol/L)**							
Quartile 1	Reference		Reference		Reference		
Quartile 2	−0.02(−0.07, 0.03)	0.336	−0.02(−0.09, 0.05)	0.575	−0.02(−0.10, 0.06)	0.604	
Quartile 3	−0.07(−0.12, −0.02)	0.009	−0.05(−0.12, 0.03)	0.197	−0.09(−0.17, −0.02)	0.015	
Quartile 4	−0.10(−0.15, −0.05)	<0.001	−0.10(−0.18, −0.03)	0.006	−0.10(−0.18, −0.03)	0.004	
Trend test		<0.001		0.001		0.002	
Increase per SD	−0.03(−0.05, −0.02)	<0.001	−0.04(−0.06, −0.01)	0.006	−0.03(−0.06, −0.01)	0.011	0.845
**LDL-C (mmol/L)**							
Quartile 1	Reference		Reference		Reference		
Quartile 2	−0.02(−0.16, 0.11)	0.732	0.01(−0.18, 0.19)	0.962	−0.01(−0.21, 0.19)	0.903	
Quartile 3	0.06(−0.08, 0.20)	0.420	0.09(−0.13, 0.30)	0.427	0.05(−0.15, 0.24)	0.626	
Quartile 4	0.06(−0.07, 0.20)	0.356	0.14(−0.07, 0.35)	0.180	−0.01(−0.20, 0.17)	0.877	
Trend test		0.167		0.079		0.997	
Increase per SD	0.04(−0.01, 0.09)	0.120	0.08(0.01, 0.15)	0.037	−0.00(−0.06, 0.06)	0.964	0.304

*SD, standard deviation*.

a *Adjusted for age, gender, BMI, educational level, smoking, drinking, household income*.

b *Adjusted for age, BMI, educational level, smoking, drinking, household income*.

c *The interaction between ESRα methylation and gender*.

### Association Between Lipid Metabolism and T2DM

The association between lipid metabolism and T2DM is presented in Table 3. The risk of T2DM shown an upward trend with increasing quartiles of TG whether gender stratification was performed (all *P* for trend < 0.05). In continuous analysis, after adjusting for potential confounding factors, each unit SD increase in TG was associated with an increase of 43%, 36% and 49% in the risk of T2DM in all population, men and women respectively (all *P* < 0.05).

**Table 3:** Association between lipid metabolism and T2DM.

**Lipid metabolism** **(mmol/L)**	**T2DM**						
	**All**^****^a^****^		**Men**^****^b^****^		**Women**^****^b^****^		***P* for interaction^**^c^**^**
	**OR (95% *CI*)**	***P***	***OR* (95% *CI*)**	***P***	**OR (95% *CI*)**	***P***	
**TG**							
Quartile 1	Reference		Reference		Reference		
Quartile 2	1.65(1.02, 2.69)	0.42	2.33(1.06, 5.11)	0.035	1.32(0.68, 2.54)	0.413	
Quartile 3	1.52(1.05, 2.21)	0.027	1.53(0.86, 2.74)	0.149	1.41(0.85, 2.33)	0.179	
Quartile 4	2.30(1.59, 3.33)	<0.001	2.64(1.49, 4.65)	0.001	1.91(1.16, 3.14)	0.011	
Trend test		<0.001		<0.001		0.011	
Increase per SD	1.43(1.25, 1.64)	<0.001	1.36(1.13, 1.64)	0.001	1.49(1.21, 1.83)	<0.001	0.549
**TC**							
Quartile 1	Reference		Reference		Reference		
Quartile 2	1.18(0.76, 1.83)	0.468	1.42(0.75, 2.70)	0.284	0.74(0.38, 1.43)	0.368	
Quartile 3	1.26(0.82, 1.95)	0.293	1.17(0.61, 2.24)	0.630	1.17(0.63, 2.16)	0.625	
Quartile 4	1.39(0.90, 2.13)	0.134	1.37(0.72, 2.62)	0.335	1.21(0.66, 2.22)	0.530	
Trend test		0.121		0.467		0.486	
Increase per SD	1.15(0.99, 1.33)	0.061	1.03(0.84, 1.29)	0.722	1.18(0.96, 1.45)	0.121	0.426
**HDL-C**							
Quartile 1	Reference		Reference		Reference		
Quartile 2	0.90(0.60, 1.34)	0.593	0.82(0.46, 1.45)	0.489	0.98(0.54, 1.77)	0.941	
Quartile 3	0.81(0.54, 1.24)	0.331	0.66(0.35, 1.22)	0.184	0.99(0.54, 1.81)	0.977	
Quartile 4	0.91(0.59, 1.41)	0.679	0.47(0.22, 0.97)	0.041	1.55(0.86, 2.81)	0.148	
Trend test		0.478		0.010		0.168	
Increase per SD	0.99(0.85, 1.15)	0.862	0.75(0.58, 0.97)	0.028	1.18(0.97, 1.44)	0.097	0.019
**LDL-C**							
Quartile 1	Reference		Reference		Reference		
Quartile 2	0.58(0.36, 0.92)	0.020	0.39(0.19, 0.81)	0.011	0.70(0.36, 1.36)	0.290	
Quartile 3	0.79(0.51, 1.22)	0.282	0.78(0.42, 1.43)	0.423	0.55(0.28, 1.09)	0.085	
Quartile 4	0.87(0.57, 1.34)	0.527	0.69(0.36, 1.32)	0.262	0.98(0.53, 1.82)	0.957	
Trend test		0.923		0.532		0.976	
Increase per SD	0.94(0.81, 1.10)	0.452	0.89(0.70, 1.12)	0.318	0.95(0.76, 1.17)	0.614	0.585

*SD, standard deviation*.

a *Adjusted for age, gender, BMI, educational level, smoking, drinking, household income*.

b *Adjusted for age, BMI, educational level, smoking, drinking, household income*.

c *The interaction between lipid metabolism and gender*.

In addition, the risk of T2DM shown a downward trend with increasing levels of HDL-C in men (*P* for trend <0.05). In continuous analysis, we observed a decrement of 25% in the risk of T2DM with each unit SD increase of HDL-C levels in men. Furthermore, the interactive effect between HDL-C and gender on the risk of T2DM was evaluated, and significant association was observed.

### The Role of *ESRα* Methylation in the Association Between Lipid Metabolism and T2DM

We did not find any association between *ESR*α promoter methylation and FPG (Supplementary Table 3) or T2DM (Table 4). After stratifying the population into two groups by the *ESR*α promoter methylation level, we found that the risk of T2DM in participants with lower methylation (≤4.07%) were more susceptible to HDL-C (*P* for interaction = 0.030), as manifested by a decreased of 22% in the risk of T2DM with the increment of each unit SD in HDL-C concentration (Table 5).

**Table 4:** Association between *ESR*α methylation and T2DM.

***ESRα* methylation (%)**	**All^a^**		**Men^b^**		**Women^b^**	
	**OR (95% CI)**	***P***	**OR (95% CI)**	***P***	**OR (95% CI)**	***P***
Quartile 1	Reference		Reference		Reference	
Quartile 2	1.20 (0.77, 1.86)	0.429	1.53 (0.80, 2.93)	0.200	1.14 (0.60, 2.17)	0.693
Quartile 3	1.01 (0.64, 1.58)	0.974	0.94 (0.47, 1.88)	0.851	1.05 (0.57, 1.96)	0.868
Quartile 4	0.94 (0.59, 1.49)	0.778	1.35 (0.64, 2.84)	0.427	0.70 (0.38, 1.30)	0.259
Trend test		0.276		0.896		0.105
Increase per SD	0.95 (0.81, 1.11)	0.486	1.10 (0.86, 1.41)	0.440	0.82 (0.67, 1.02)	0.070

*SD, standard deviation*.

a *Adjusted for age, gender, BMI, educational level, smoking, drinking, household income*.

b *Adjusted for age, BMI, educational level, smoking, drinking, household income*.

**Table 5:** Interactive effects of *ESR*α methylation and lipid metabolism indexes on T2DM.

**Lipid metabolism**	**Groups**	**T2DM**^****^a^****^		
		***OR* (95%CI)**	***P***	***P*-interaction^**^b^**^**
TG	*ESRα* methylation			0.455
	≤4.07%	1.34(1.08, 1.65)	0.007	
	**>**4.07%	1.67(1.34, 2.09)	<0.001	
TC	*ESRα* methylation			0.757
	≤4.07%	1.10(0.89, 1.36)	0.390	
	**>**4.07%	1.22(0.98, 1.53)	0.082	
HDL-C	*ESRα* methylation			0.030
	≤4.07%	0.78(0.61, 1.00)	0.046	
	**>**4.07%	1.04(0.83, 1.31)	0.736	
LDL-C	*ESRα* methylation			0.273
	≤4.07%	1.02(0.81, 1.28)	0.878	
	**>**4.07%	0.88(0.69, 1.13)	0.322	

a *Adjusted for age, gender, BMI, educational level, smoking, drinking, household income*.

b *The interaction between lipid metabolism and ESRα methylation*.

## Discussion

In the current study, we explored the demographic information of the demographic information and found that the prevalence of T2DM in men is significantly lower than in women. Several previous studies have also observed gender differences in the prevalence of T2DM (24–26). Among them, a large-scale epidemiological survey found that the risk of T2DM in male farmers in rural areas is lower than in female farmers (26). The physical activity has been reported to mitigate the impaired glucose tolerance caused by unhealthy lifestyle (such as sleep loss) (27) and prevent the occurrence of T2DM (28). Farmers were recruited as participants in our study. And the level of physical activity of male farmers is significantly higher than that of female farmers (29), which may lead to a lower prevalence of T2DM in men.

Besides, the association between *ESR*α promoter methylation and lipid metabolism was investigated. And negative correlations were observed between *ESR*α promoter methylation and HDL-C levels, whether gender stratification was performed. Considering that the *ESR*α promoter methylation can suppress the protein expression of ESRα (30), we speculate that the ESRα levels may relate positively to the HDL-C levels in adults. These findings are similar to the previous studies (12, 31). The expression of LDL receptor has been reported to depend on tyrosine kinase and protein kinase C activation, both signal pathways could be activated by estrogen (32). Additionally, as a regulator of LDL-C metabolism, *ESR*α can affect the gene expression of LDL-C receptors (33). Whereas Knopp et al. (34) observed a lesser change in low-density lipoprotein in women than men with high-carbohydrate or high-fat feeding. Here, we only observed a positive correlation between *ESR*α promoter methylation and LDL-C levels in men, whereas no association was observed in women, further suggesting that LDL-C may be affected by genetic factors in a gender-specific manner.

The concentrations of LDL-C in patients with T2DM are generally not significantly different from those in non-diabetic patients (35). Similarly, no significant association between the levels of LDL-C and the risk of T2DM was observed in the present study. This may be caused by the the management of LDL-C or the reduction of LDL-C catabolism in patients (8, 36). Besides, we here found that the risk of T2DM was positively associated with TG whether gender stratification was performed. Besides, we observed a negative correlation between HDL-C levels and the risk of T2DM in men, instead of women. As revealed in the previous study (37), the dominant lipid abnormality in diabetes is hypertriglyceridemia, which is commonly associated with a reduction in HDL-C. Our findings are similar to the previous study, suggesting that TG and HDL-C may be closely related to the risk of T2DM. Hanai et al. (38) found that the levels of HDL-C were associated with the progression of diabetic kidney disease in men but not in women. In addition, a greater difference in those with diabetes compared with those without diabetes were observed in women than in men for HDL-C (39). Combining these findings, we speculate that there may be gender difference in relationship between HDL-C and T2DM, and a relatively small alteration in HDL-C may trigger the occurrence of T2DM in male.

A previous study found that the level of *ESR*α promoter methylation in decidual tissue of Germans with gestational diabetes mellitus (GDM) is higher than that of Germans without GDM (40), indicating that *ESR*α promoter methylation may increase the risk of GDM. While in this study, we did not observe any association between *ESR*α promoter methylation and FPG or the risk of T2DM. These inconsistencies may be due to the obvious difference races and different types of diabetes mellitus. Notably, many reproducible studies found that the polymorphisms in the same site (rs1801282 in *PPARG* gene) is not significantly associated with the risk of GDM, while it can elevate the risk of T2DM (41), suggesting that the same genetic changes may have different associations with different types of diabetes mellitus (42, 43). Finally, we observed a significant interactive effect of *ESR*α promoter methylation and HDL-C on the risk of T2DM, indicating that the level of *ESR*α methylation may modify the association between HDL-C and the risk of T2DM, and the risk of T2DM in participants with lower *ESR*α methylation is more susceptible to the alteration of HDL-C. Different DNA fragments located in the same gene may have different methylation levels and thus result in different biological effects (44). Consequently, further comprehensive methylation sequencing in large population may provide more clues for the pathogenesis of T2DM.

## Conclusion

In summary, lipid metabolism was associated with the levels of *ESR*α promoter methylation and the risks of T2DM. Additionally, *ESR*α promoter methylation can modify the association of HDL-C and T2DM.

## Data Availability Statement

The raw data supporting the conclusions of this article will be made available by the authors, without undue reservation.

## Ethics Statement

The studies involving human participants were reviewed and approved by The project was approved by the Institutional Review Board of Zhengzhou University. The patients/participants provided their written informed consent to participate in this study.

## Author Contributions

LW, HH, WL, CW, and YB designed the research. GZ, XL, RS, and ZL collected the data. RS and LL performed the experiments. GZ and LL analyzed the data and wrote the manuscript. YB revised the language/article. All authors read and approved the final manuscript.

## Conflict of Interest

The authors declare that the research was conducted in the absence of any commercial or financial relationships that could be construed as a potential conflict of interest. The reviewer DH declared a shared affiliation, with no collaboration, with the authors to the handling editor at the time of the review.

## Abbreviations

*ESR*α: estrogen receptor alpha
T2DM: type 2 diabetes mellitus
HDL-C: high density lipoprotein cholesterol
LDL-C: low density lipoprotein cholesterol
TG: triglyceride
TC: total cholesterol
FPG: fasting plasma-glucose.
