# Supplementary material for: ESRα Promoter Methylation May Modify the Association Between Lipid Metabolism and Type 2 Diabetes in Chinese Farmers
Source: Front Public Health. 2021 Mar 4;9:578134. doi: 10.3389/fpubh.2021.578134 (PMC7969800; doi:10.3389/fpubh.2021.578134)
Supplement: Supplementary file 1 [file Data_Sheet_1.docx]

**Supplementary Materials**

***ESRα* promoter methylation may modify the association between lipid metabolism and type 2 diabetes in Chinese farmers**

**Tables of Contents**

**Table S1****.** The Quartiles of *ESRα* methylation and lipid metabolism.

**Table S2.** Differences in biomarkers among different age groups.

**Table S3.** Association between *ESRα* methylation and FPG.

**Table S1.** The Quartiles of *ESRα* methylation and lipid metabolism.

| Variables | Range | | | |
| --- | --- | --- | --- | --- |
|  | Quartile 1 | Quartile 2 | Quartile 3 | Quartile 4 |
| All |  |  |  |  |
| *ESRα* methylation (%) | ≤1.37 | 1.38-1.84 | 1.85-2.46 | ＞2.47 |
| TG (mmol/L) | ≤0.66 | 0.67-1.04 | 1.05-1.62 | ＞1.63 |
| TC (mmol/L) | ≤4.01 | 4.02-4.57 | 4.58-5.25 | ＞5.26 |
| HDL-C (mmol/L) | ≤3.36 | 3.37-3.99 | 4.00-4.72 | ＞4.73 |
| LDL-C (mmol/L) | ≤2.74 | 2.75-3.31 | 3.32-3.98 | ﹥3.99 |
| Men |  |  |  |  |
| *ESRα* methylation (%) | ≤1.36 | 1.37-1.82 | 1.83-2.35 | ﹥2.36 |
| TG (mmol/L) | ≤0.66 | 0.67-1.06 | 1.07-1.68 | ﹥1.69 |
| TC (mmol/L) | ≤3.96 | 3.97-4.51 | 4.52-5.19 | ﹥5.20 |
| HDL-C (mmol/L) | ≤3.32 | 3.33-3.92 | 3.93-4.62 | ﹥4.63 |
| LDL-C (mmol/L) | ≤2.68 | 2.69-3.26 | 3.27-3.90 | ﹥3.91 |
| Women |  |  |  |  |
| *ESRα* methylation (%) | ≤1.38 | 1.39-1.87 | 1.88-2.59 | ﹥2.60 |
| TG (mmol/L) | ≤0.65 | 0.66-1.01 | 1.02-1.56 | ﹥1.57 |
| TC (mmol/L) | ≤4.06 | 4.07-4.62 | 4.63-5.31 | ﹥5.32 |
| HDL-C (mmol/L) | ≤3.39 | 3.40-4.05 | 4.06-4.78 | ﹥4.79 |
| LDL-C (mmol/L) | ≤2.79 | 2.80-3.38 | 3.39-4.09 | ﹥4.10 |

**Table S2.** Differences in biomarkers among different age groups.

| Biomarkers | Participants | | | *F/Z* | *P* |
| --- | --- | --- | --- | --- | --- |
|  | ≤50 years | 51-60 years | >60 years |  |  |
| *n* | 397 | 361 | 385 |  |  |
| *ESRα* methylation (%) | 4.05±1.87 | 4.60±2.20 | 4.99±2.44 | 16.622 | <0.001 |
| FPG (mmol/L) | 4.74(4.23,5.41) | 5.05(4.44,5.90) | 5.26(4.62,6.24) | 44.321 | <0.001 |
| TG (mmol/L) | 1.90±1.58 | 1.85±1.41 | 1.75±1.17 | 1.096 | 0.335 |
| TC (mmol/L) | 4.54±1.01 | 4.67±0.96 | 4.66±1.00 | 1.917 | 0.148 |
| HDL-C (mmol/L) | 1.24±0.29 | 1.25±0.30 | 1.22±0.31 | 0.693 | 0.500 |
| LDL-C (mmol/L) | 2.47±0.75 | 2.63±0.77 | 2.66±0.77 | 6.806 | 0.001 |

^a^ Data are expressed as the mean ± SD or Median (P25, P75) for continuous variables and *n* (%) for categorical variables.

**Table S3.** Association between *ESRα* methylation and FPG.

| *ESRα* methylation (%) | All ^a^ | |  | Men ^b^ | |  | Women ^b^ | |
| --- | --- | --- | --- | --- | --- | --- | --- | --- |
|  | *β* (95% *CI*) | *P* |  | *β* (95% *CI*) | *P* |  | *β* (95% *CI*) | *P* |
| Quartile 1 | Reference |  |  | Reference |  |  | Reference |  |
| Quartile 2 | 0.10 (−0.51, 0.71) | 0.753 |  | −0.07 (−1.04, 0.89) | 0.885 |  | 0.29 (−0.47, 1.04) | 0.453 |
| Quartile 3 | −0.08 (−0.55, 0.39) | 0.742 |  | −0.03 (−0.67, 0.62) | 0.939 |  | −0.37 (−1.08, 0.35) | 0.309 |
| Quartile 4 | −0.05 (−0.11, 0.02) | 0.163 |  | −0.06 (−0.17, 0.05) | 0.255 |  | −0.05 (−0.13, 0.03) | 0.204 |
| Trend test |  | 0.709 |  |  | 0.075 |  |  | 0.293 |
| Increase per SD | −0.03 (−0.11, 0.05) | 0.480 |  | −0.07 (−0.21, 0.06) | 0.282 |  | −0.02 (−0.12, 0.08) | 0.691 |

Abbreviations: SD, standard deviation.

^a^ Adjusted for age, gender, BMI, educational level, smoking, drinking, household income.

^b^ Adjusted for age, BMI, educational level, smoking, drinking, household income.
